# Melanoma Severity at Presentation and Prior Skin Cancer Screening: A Survey Study of Surgical Oncology Patients

**DOI:** 10.1155/jskc/3520345

**Published:** 2026-09-15

**Authors:** Arnar B. Ingason, Venkatesa P. Muruganandam, Melanie Bui, Jessica Cintolo-Gonzalez

**Affiliations:** ^1^ Division of Surgical Oncology, Larner College of Medicine, University of Vermont, Burlington, Vermont, USA, uvm.edu; ^2^ Division of Dermatology, Larner College of Medicine, University of Vermont, Burlington, Vermont, USA, uvm.edu

**Keywords:** dermatologic screening, full-body skin examination, malignant melanoma, screening

## Abstract

**Introduction:**

The effectiveness of population‐based melanoma screening remains uncertain. However, most melanomas continue to present outside of skin cancer screening, underscoring the need to better understand and serve unscreened populations.

**Methods:**

All patients presenting to surgical oncology at a rural tertiary referral center with newly diagnosed invasive melanoma from April 2020 to January 2021 were administered a survey to assess history of a skin cancer screening examination and risk factors for melanoma. Additional demographic and clinical characteristics were abstracted from the electronic medical record. The exposure of interest was dermatological skin cancer screening examination within the past 12 months. The primary outcome was pathological tumor staging. This was assessed using ordinal logistic regression.

**Results:**

Forty patients presented to surgical oncology with a newly diagnosed melanoma. Thirty patients (75%) reported no skin cancer screening examination within the past 12 months. Patients who had screening were more likely to report prior indoor tanning use (70% [7/10] vs. 23% [7/30], OR 7.20, 95% CI 1.25–55.24, *p* = 0.02) and a personal history of nonmelanoma skin cancer (60% [6/10] vs. 13% [4/30], OR 9.00, 95% CI 1.44–68.66 *p* = 0.007). Remaining baseline characteristics were similar. Patients who had screening presented with a significantly lower T stage (OR 0.24, 95% CI 0.05–0.91, *p* = 0.04). No patients who had screening presented with lymph node metastasis compared with 20% of patients (6/30) who did not undergo screening. No patients in our cohort presented with distant metastasis.

**Conclusions:**

Most melanomas presenting to surgical oncology occurred in patients without recent screening and were more advanced at presentation. These findings highlight the importance of maintaining rapid access to dermatologic evaluation for patients who do not participate in routine screening, as they represent the majority of clinically significant melanoma presentations.

**Key Points**
• Question: Is skin cancer screening associated with lower cancer staging?
• Findings: In this cross‐sectional study of 40 patients with newly diagnosed malignant melanoma, patients who had skin cancer screening within the past 12 months presented with a significantly lower tumor stage (OR 0.24, 95% CI 0.05–0.91, *p* = 0.04). No patients who had skin cancer screening presented with lymph node metastasis compared with 20% of patients (6/30) who did not undergo screening.
• Meaning: Most melanomas presenting to surgical oncology occurred in patients without recent screening and were more advanced at presentation.

## 1. Introduction

Melanoma accounts for approximately 1% of all skin cancers but disproportionately contributes to skin cancer mortality [1, 2]. In the United States, more than 8000 deaths occur annually, and survival varies markedly by stage, exceeding 99% for localized disease but decreasing to approximately 35% for distant‐stage disease [3, 4]. These characteristics have made melanoma an attractive candidate for population‐based screening.

Evidence supporting routine skin cancer screening remains mixed. Population‐based screening in Schleswig‐Holstein, Germany, was associated with a 48% reduction in melanoma mortality, and subsequent analyses demonstrated that melanomas detected by skin cancer screening were associated with lower mortality (adjusted hazard ratio [aHR], 0.62; 95% confidence interval [CI], 0.48–0.80) and fewer advanced‐stage presentations [5, 6]. However, these effects were not sustained, and later studies failed to demonstrate a consistent population‐level benefit [5]. Similarly, a large study of 595,799 patients in US primary care settings found that screening increased detection of thin melanomas without a corresponding increase in detection of thicker lesions [7].

Accordingly, the 2023 US Preventive Services Task Force guideline concludes that evidence is insufficient to recommend routine skin cancer screening for asymptomatic individuals [8]. While prior work has focused on identifying populations most likely to benefit from screening and expanding access to screening, less attention has been directed toward patients who do not engage in routine screening. These individuals may represent a substantial proportion of melanoma cases encountered in surgical oncology.

These observations suggest that improving melanoma outcomes may depend not only on screening uptake but also on ensuring timely access to dermatologic evaluation for patients with new or changing lesions who present outside of routine screening.

The objective of this study was to quantify the proportion of patients presenting to surgical oncology with invasive melanoma who had undergone a skin cancer screening examination within the prior 12 months and to compare the stage at presentation between screened and unscreened patients.

## 2. Methods

### 2.1. Patient Population

All patients aged ≥ 18 years presenting to surgical oncology at a rural tertiary referral center with newly diagnosed malignant melanoma from April 2020 to January 2021 were eligible for study inclusion. Patients were excluded if they had a personal history of melanoma or if they were aged < 18 years. Following informed consent, patients were administered a survey comprising risk factors for melanoma, prior melanoma education, and skin screening history. The survey was created specifically for this study and had not been previously validated. Additional demographic and clinical characteristics and staging were abstracted from the electronic medical record. This study was approved by the Institutional Review Board of the University of Vermont (#00000624).

### 2.2. Exposure and Outcomes

The exposure of interest was dermatological skin cancer screening within the past 12 months. Dermatological skin cancer screening was defined as a full‐body skin examination performed by a qualified medical clinician. The primary endpoints were pathological tumor (*T*) and nodal (N) staging, as defined by the eighth edition of the American Joint Committee on Cancer TNM classification system [9]. Patients were followed from the time of diagnosis to the time of definitive treatment.

### 2.3. Statistical Analysis

Statistical analysis was performed in *R*, Version 4.5.1, using RStudio (R Foundation for Statistical Computing, Austria). Statistical tests were two‐tailed, and a *p* value < 0.05 was considered statistically significant. The Mann–Whitney *U* test and Fisher’s exact test were used to compare continuous and categorical variables, respectively. T‐staging was compared using univariate ordinal logistic regression. The proportional odds assumption was evaluated using the Brant test, with no evidence of violation (*p* = 0.93).

## 3. Results

Overall, 40 patients presented to surgical oncology with their first primary cutaneous melanoma and were enrolled in our study. Of those, thirty (75%) lacked skin cancer screening within the past year. Patients who had skin cancer screening were more likely to report prior indoor tanning use (70% [7/10] vs. 23% [7/30], OR 7.20, 95% CI 1.25–55.24, *p* = 0.02) and a personal history of nonmelanoma skin cancer (60% [6/10] vs. 13% [4/30], OR 9.00, 95% CI 1.44–68.66 *p* = 0.007). Remaining baseline characteristics were similar (Table 1). Time from discovery to diagnosis (30 days [interquartile range (IQR) 14–144 days] vs. 107 days [IQR 14–184 days, *p* = 0.51) and from diagnosis to definitive treatment (28 days [IQR 23–41 days] vs. 23 days [IQR 21–34 days], *p* = 0.44) did not differ significantly between patients who underwent skin cancer screening and those who did not.

**TABLE 1:** Comparison of baseline characteristics between patients with newly diagnosed melanoma who underwent skin cancer screening within the past year and those who did not.

Variable	No screening (*n* = 30)	Screening (*n* = 10)	*p* value
Age	63.5 (51.0–69.0)	58.0 (53.3–66.5)	0.95
Sex (male)	21 (70.0)	4 (40.0)	0.30
Family history of skin cancer			0.55
Unknown, not specified	1 (3.3)	0 (0.0)	
No	21 (70.0)	9 (90.0)	
Yes	8 (26.7)	1 (10.0)	
Primary payer information (%)			0.25
Medicaid	5 (16.7)	0 (0.0)	
Medicare	9 (30.0)	3 (30.0)	
Private insurance	16 (53.3)	6 (60.0)	
Unknown	0 (0.0)	1 (10.0)	
Distance to University of Vermont Medical Center	73.0 (47.8–91.3)	42.5 (27.0–69.3)	0.15
Education level			0.16
Unknown, not specified	9 (30.0)	0 (0.0)	
High school or equivalent	7 (23.3)	3 (30.0)	
College	12 (40.0)	5 (50.0)	
Graduate level	2 (6.7)	2 (20.0)	
Marital status			1.00
Married/legal partnership	19 (63.3)	7 (70.0)	
Committed relationship	3 (10.0)	1 (10.0)	
Single	8 (26.7)	2 (20.0)	
> 3 months per year spent in another region	2 (6.7)	2 (20.0)	0.26
Majority of childhood in a different country	7 (23.3)	5 (50.0)	0.13
Primary site location			0.79
Head and neck	8 (26.7)	4 (40.0)	
Trunk	14 (46.7)	3 (30.0)	
Upper extremity or shoulder	4 (13.3)	2 (20.0)	
Lower extremity	4 (13.3)	1 (10.0)	
Lesion first discovered by self	17 (56.7)	6 (60.0)	1.00
Smoking status			0.45
Current smoker	6 (20.0)	1 (10.0)	
Former smoker	8 (26.7)	1 (10.0)	
Never smoker	16 (53.3)	8 (80.0)	
History of blistering or peeling sunburns	18 (60.0)	4 (40.0)	0.30
History of indoor tanning	7 (23.3)	7 (70.0)	0.02
Prior diagnosis of nonmelanoma skin cancer	4 (13.3)	6 (60.0)	0.007

*Note:* Data are presented as *n* (%) or median (IQR).

**FIGURE 1:**
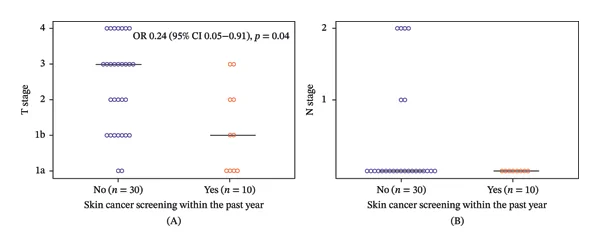
Scatter plot demonstrating differences in (A) tumor (T) staging and (B) nodal (N) staging (as defined by the eighth edition of the American Joint Committee on Cancer TNM classification system) for patients with melanoma depending on whether they underwent skin cancer screening within the past year or not. Bars represent the median stage for each group.

Patients who had skin cancer screening presented with a significantly lower T stage (OR 0.24, 95% CI 0.05–0.91, *p* = 0.04, Figure 1A). No patients who had skin cancer screening presented with lymph node metastasis compared with 20% of patients (6/30) who did not undergo screening (Figure 1B). No patients in our cohort presented with distant metastasis.

## 4. Discussion

In this study, 75% of patients presenting to surgical oncology with melanoma had not undergone recent dermatologic skin cancer screening, and these patients presented with more advanced disease. While screening was associated with earlier‐stage disease, only a minority of patients had undergone recent screening. Screening participation appears to be concentrated among individuals with some, but not all, recognized risk factors, whereas the majority of melanomas arise in patients outside of these pathways. These findings suggest that expanding screening efforts alone may not substantially alter the population burden of clinically aggressive melanoma, as those at highest risk of advanced presentation may remain unscreened. Interventions targeting screening uptake may not reach the relevant population when and where most aggressive melanomas arise.

Current national guidelines do not recommend routine skin cancer screening for asymptomatic patients [8, 10]. In this study, screening history was associated with thinner melanomas and an earlier stage at presentation. Skin cancer screening was also associated with a personal history of nonmelanoma skin cancer and indoor tanning. National guidelines support routine skin cancer screening examinations for patients with a history of skin cancer; therefore, this finding is consistent with recommended practice. Indoor tanning is a well‐established risk factor for early‐onset skin cancer including melanoma [11], and its association with recent skin cancer screening is not unexpected. One might also have expected higher rates of other risk factors, including male sex, smoking, or a history of sunburns among those undergoing screening. Instead, we observed a trend toward the opposite, which may reflect differences in health‐seeking behaviors between the two groups.

When discussing the effects of implementing routine skin cancer screening at a population level, one must also consider the effects on resource allocation. Expanding screening to individuals with less specific risk factors may strain resources and limit timely access for higher‐risk patients including those presenting with suspicious lesions, which is how most advanced melanomas present.

A key implication of these findings is the importance of timely access to dermatologic evaluation for patients who do not engage in routine screening. Patients who do not or cannot seek routine skin cancer screening examinations represent the majority of melanoma cases presenting to surgical oncology, are more likely to present with nodal disease, and do not experience delays once within the treatment system. Therefore, a critical modifiable interval may represent the time from lesion recognition to specialist evaluation [12].

These findings support a complementary strategy that prioritizes rapid‐access dermatology pathways for concerning lesions, efficient triage systems, and preservation of appointment availability for symptomatic patients, in addition to targeted skin cancer screening of high‐risk populations [12, 13]. This approach acknowledges that not all patients will participate in routine screening and focuses on optimizing outcomes within real‐world care‐seeking behaviors.

Our study must be interpreted in the context of several limitations. First, the sample size of the current study was small. Second, the use of a survey to assess recent history of skin cancer screening introduces the risk of recall bias. However, the magnitude of this risk is likely mitigated by the fact that only patients with confirmed melanoma diagnosis were included. Third, there may have been an element of selection bias related to both which patients were referred to our surgical oncology service and who underwent skin cancer screening. For example, patients with metastatic disease or patients who were deemed poor surgical candidates may not have been referred for surgical evaluation. Similarly, patients with thin melanomas may have been managed exclusively by a dermatologist. Fourth, the study was conducted at a rural tertiary center, as such, it is unclear whether our results are generalizable to larger urban areas. Fifth, our study was conducted during the COVID‐19 pandemic, which may have affected skin cancer screening patterns. Lastly, long‐term outcomes were not assessed.

In conclusion, our study found that the majority of invasive melanomas requiring surgical oncology care arose in patients who were not engaged in screening pathways. Additionally, skin cancer screening was associated with lower cancer staging. However, it should be emphasized that this association does not necessarily equate causality. Large prospective studies are required to confirm our findings. It is also worth emphasizing that improving melanoma outcomes may depend not only on screening efforts but also on ensuring rapid dermatologic access for unscreened patients, who represent the largest and highest‐risk group at presentation.

## Funding

The study was supported by a training grant from the Northern New England Clinical Oncology Society.

## Consent

Individual patient consent was acquired before patient enrollment in the study.

## Conflicts of Interest

The authors declare no conflicts of interest.

## Data Availability

Data are available from the corresponding author upon reasonable request.
